# On the ability of molecular dynamics simulation and continuum electrostatics to treat interfacial water molecules in protein-protein complexes

**DOI:** 10.1038/srep38259

**Published:** 2016-12-01

**Authors:** Guillaume Copie, Fabrizio Cleri, Ralf Blossey, Marc F. Lensink

**Affiliations:** 1 University Lille, CNRS, UMR8520 IEMN, Lille, F-59000, France; 2 University Lille, CNRS, UMR8576 UGSF, Lille, F-59000, France

## Abstract

Interfacial waters are increasingly appreciated as playing a key role in protein-protein interactions. We report on a study of the prediction of interfacial water positions by both Molecular Dynamics and explicit solvent-continuum electrostatics based on the Dipolar Poisson-Boltzmann Langevin (DPBL) model, for three test cases: (i) the barnase/barstar complex (ii) the complex between the DNase domain of colicin E2 and its cognate Im2 immunity protein and (iii) the highly unusual anti-freeze protein Maxi which contains a large number of waters in its interior. We characterize the waters at the interface and in the core of the Maxi protein by the statistics of correctly predicted positions with respect to crystallographic water positions in the PDB files as well as the dynamic measures of diffusion constants and position lifetimes. Our approach provides a methodology for the evaluation of predicted interfacial water positions through an investigation of water-mediated inter-chain contacts. While our results show satisfactory behaviour for molecular dynamics simulation, they also highlight the need for improvement of continuum methods.

Water is essential to all life that we know of. Its omnipresence in biological processes is generally assumed and as a consequence its molecular structure is often ignored when devising theories about the molecular details of those processes. At a coarse-grained scale, solvent effects are responsible for phenomena such as electrostatic screening, where they effectively reduce the electrostatic field of the molecules they surround^1^, and the hydrophobic effect^2^, which leads to self-assembly or aggregation of nonpolar molecules in an attempt to minimize their interaction with water^3^. Insights in the molecular details of the aggregation reveal that the hydrophobic effect is an entropy-driven process, which is fueled by an increased freedom for the water molecules to engage in hydrogen bonding. The difference in hydrogen-bonding behaviour between bulk water and water molecules at the water-detergent interface have been made evident by time-resolved vibrational spectroscopy, showing two distinct but exchanging populations^4^.

Water molecules also solvate protein structures. Due to the polar nature of the protein surface, the change in properties between the solvation shell and bulk water is smaller as opposed to the detergent-water interface, but nevertheless distinct: a notable difference in the water-water hydrogen bonded network can be observed. This phenomenon manifests itself in various ways in different systems, as the following short list shows. An investigation of the dynamic properties of water around simple polypeptides shows the formation of a pseudo-rigid structure around the peptide core that exhibits stronger hydrogen bonding and with longer lifetimes^5^. The formation of networks of hydration water molecules around protein structures had been described before from the investigation of cryogenic X-ray structures of bovine beta-trypsin^6^. It was found that the water network allowed exposure of the active site to bulk solvent, thereby avoiding the hampering of its protease activity. Gradients of coupled protein-water motions have also been observed near the MT1 metalloprotease active site^7^ and the so-called ice-binding plane of antifreeze proteins^8^. The water shell around the p53 core domain has been described as also consisting of two such regimes: a dynamical one, showing fast exchanges with bulk water that are unambiguously assisted by local protein motions, and a structural one that contributes to the structural integrity of the protein^9^.

Crystal structures often show crystal waters associated to the protein. An analysis of water molecules within a 5 Å shell around dimeric crystal structures has shown preferential binding to protein-protein interaction interfaces, whether these are true interfaces or crystal contacts^10^. Water unmistakably influences structure, function and stability of proteins and protein complexes^11^,^12^,^13^,^14^. The prototypical example for strong electrostatic binding, the high-affinity barnase/barstar complex, features a large amount of water molecules in its protein-protein interface, a third of which are fully buried^15^. The association between barnase and its inhibitor barstar is an extreme example of hydrophilic association, which is characterized by the anisotropy of interfacial water molecules that contribute to the association funnel^16^. The opposite extreme, hydrophobic association, exhibits a bimodal binding where hydrophobic dewetting takes place after initial long-range electrostatic attraction.

Interfacial water molecules are also crucial to both stability and specificity of colicin DNase-immunity protein complexes^17^. The complex between the DNase domain of colicin E2 and its cognate Im2 immunity protein^18^, resolved at 1.72 Å resolution at a temperature of 100K, featured as Target 47 in the CAPRI protein docking experiment^19^. With high-quality template structures of the complex available in the PDB, both cognate and non-cognate, the focus of the experiment lay in the prediction of the water positions at the protein-protein interface. It was clear from the experiment that further work in the prediction of interfacial water molecules was required. Nevertheless, the results were encouraging: several of the conserved water molecules, which define the interface hot spot, were correctly predicted, as was at least one of the water molecules responsible for the specificity for the family of complexes^19^. The more sophisticated methods employed, which often combine the use of classical empirical force fields with additional sampling and energy minimization, were found to be more successful than simpler water placement methodologies.

When studying the microscopic interaction of proteins and protein complexes with its solvating environment, molecular dynamics (MD) simulation seems the method of choice, as it not only allows to sketch a molecular picture of the interactions involved, but also provides a dynamical viewpoint. In terms of protein-water interaction, MD has been used to study the microscopic dynamics of water around unfolded proteins^20^ or to look at diffusion around intrinsically disordered proteins^21^ but it also allowed to establish the existence of coupled interactions between two distant proteins that were mediated by water^22^.

Continuum electrostatics methods, on the other hand, have so far been mainly employed for a quantification of the energetics of protein interactions. Their common assumption relies on a constant permittivity of the solvent, both in the Generalized Born (GB) approach and the Poisson-Boltzmann (PB) theory. The latter relies on the partial differential equation for the electrostatic potential *ϕ*(r).


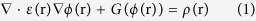


where *ε*(r) is the dielectric function, typically chosen as a constant value inside the protein (*ε* ≈ 2–4) and in water (*ε* ≈ 80); *G*(*ϕ*(r)) is a generally nonlinear function of the electrostatic potential of the mobile charges; and *ρ*(r) is the charge distribution of the fixed charges. These theories are computationally less demanding than an all-atom description of solvent effects. However, they have been found to be underperforming for large-scale simulations^23^ and unusable for protein design applications^24^, due to an underestimation of the hydrophobic forces, leading *e.g.* to burial of salt bridges. Recently, microscopic details of solvent structure have been integrated into the PB-approach leading to formulations of continuum electrostatics with explicit solvent. A simple continuum electrostatics model with explicit water is the Dipolar Poisson-Boltzmann Langevin (DPBL) model^25^,^26^. While also being a mean-field theory for the electrostatic potential *ϕ*(r) of the system, this model goes beyond the usually employed PB theory—which is also a mean-field theory–by explicitly introducing solvent molecules in the form of point dipoles. In Eq. (1) this amounts to the introduction of a dependence of the dielectric permittivity on a nonlinear function of *ϕ*(r) via


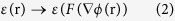


where *F* is a nonlinear function, resulting in terms of higher powers of 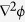 in the DPBL equations as compared to the PB equation^27^.

This approach underlies the Marcus functional employed in studies of electron transfer^28^, which is equivalent to the DPBL-model in its linearized form^29^,^30^, and was also employed in the work by Warshel and Levitt^31^. The DPBL-model lies at the basis of a dedicated solver, AquaSol^27^, built on an original PB solver^32^. It has previously been applied *e.g.* to the computation of SAXS profiles based on an extension of the AquaSol solver by the AquaSAXS module^33^, and it was also used to predict free energies of amyloid fibril aggregates^34^.

In this work, we apply both MD simulations and the DPBL model as implemented in the AquaSol server for the prediction of water positions at protein-protein interaction interfaces. We are looking at phenomena related to the motion of water molecules, and use the crystal structure as frame of reference, hence relatively short simulations suffice, as they only need to allow the water molecules to diffuse and no large-scale motions of the proteins are involved. In order to convince ourselves of the correctness of this assumption we have monitored the evolution of the so-called *f*^w^(nat) value (defined below), which for all our chosen complexes rapidly settles around a well-defined mean-value (data not shown).

As our study systems we have chosen three distinct but challenging systems to test the methodologies, which are the two complexes already discussed before: (i) the barnase/barstar complex^15^ (hereafter abbreviated by Barnase) and (ii) colicin DNase E2/Im2 protein complex^18^ or CAPRI T47^19^, and furthermore, (iii) the antifreeze protein Maxi^35^,^36^. All systems are shown in Fig. 1. Maxi is a four-helix bundle formed by head-to-tail dimerization of two 195-residue polypeptide chains. With a length of close to 15 nm and a diameter roughly one tenth of this value, it exhibits an unusual hydration of its protein core: several hundreds of water molecules form an elongated and dynamic water network – a counter example to the common cases in which water is essentially not present *within* a protein core. This case will therefore necessitate a more detailed discussion of the water molecule positions.

As the aim of this study is to investigate the performance of classical mechanical and continuum solvation methodologies in the treatment of interfacial water molecules in protein complexes, our choice reflects three different aspects of relevance for studies of water positions:

- Whereas hydrophobic association results in a protein-protein interface devoid of water molecules, the barnase/barstar complex, which has been extensively studied by computational and experimental means, is considered the prototypical example for hydrophilic association;
- E2/Im2 has been the target of the CAPRI protein docking experiment and therefore offers a direct comparison with a multitude of methodologies used for the prediction of water positions and thus easily allows the research community for additional checks;
- The Maxi protein has been chosen because the behaviour of its core water molecules falls in between the two regimes with the static interfacial waters on the one hand and bulk water on the other. It is as distinctly different from regular protein-protein interfaces as one can get, without becoming bulk water.

## Results and Discussion

**Figure 1:**
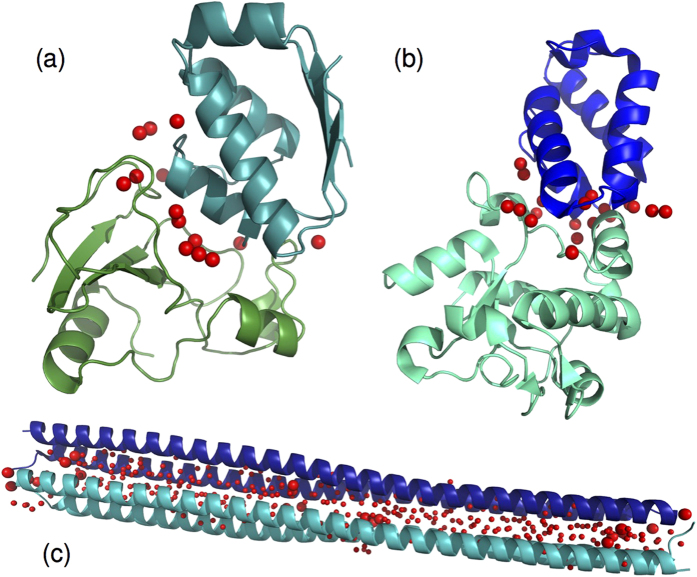
The three protein complexes. (**a**) Barnase/barstar, (**b**) E2/Im2, and (**c**) Maxi. Individual protein chains are colored differently. Red spheres indicate interfacial water molecules for the three systems. The smaller red spheres indicate additional core water molecules for Maxi (see text for definition).

**Figure 2:**
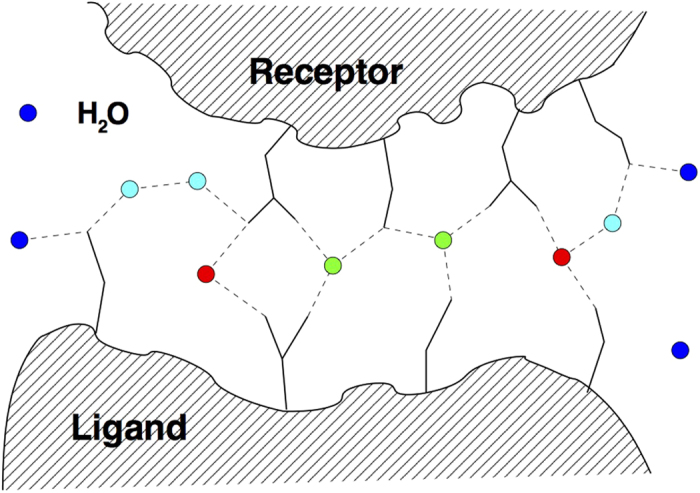
Schematic illustration of water-mediated inter-chain contacts at the interface of a protein-protein complex. Water molecules are indicated as colored circles, with red water molecules engaging in one, and green waters in multiple water-mediated contacts. Blue surface waters are bound to a single one or none of the entities (ligand or receptor) and do not contribute to the water-mediated contact list. Cyan water molecules are engaged in water-mediated ligand-receptor contacts mediated by two water molecules.

We first need to clarify the concepts that we used to characterize the different water molecules in the systems. We distinguish between:

1. *Water mediated contacts and interfacial waters.* This notion refers to the water molecules that are found in the overlap of two water shells considered around both the receptor and the ligand, hence the waters are shared by both. There are two measures that can be used for the waters within this shell: the first is the recall of native water positions, which is the ratio of predicted waters within a certain distance to those in the PDB template within this overlap region. The second is the recall of water-mediated ligand-receptor contacts *f*^w^(nat), which we use here. A contact occurs when any two (heavy) atoms of a ligand and receptor residue pair are found within a distance of 3.5 Å or less from the same water molecule, see Fig. 2; this definition is the same as in ref. 19, where it has also been shown that these two measures correlate. As Fig. 2 also shows, the number of such contacts can be larger than the actual number of water molecules.
2. *Associated waters.* These water positions refer to the water molecules that are found in the water shells surrounding the proteins but exclude the interfacial waters.
3. *Core waters.* We introduce this notion for the discussion of the Maxi complex as it contains a large number of waters between the chains. Core waters are those that were shared by the water shells around each of the four chains making up the complex. We define a water molecule to be in the core of the protein if the distance between its oxygen atom and at least one of the protein heavy atoms of both *α*-helices is less than 1.1 nm. If the interfacial water definition were used, the Maxi complex would only contain a 22 of such waters (see also Fig. 1c).
4. *Bulk waters.* These are the water molecules that are not influenced by the presence of the complex.

Table 1 summarizes the setup of the MD simulations and the computed interfacial waters and water-mediated contacts for the three complexes. An interfacial water is defined as being in contact (distance less than or equal to 3.5 Å) with both protein chains (ligand and receptor) simultaneously. A water-mediated contact is a ligand-receptor contact running over an interface water molecule. The reference number of contacts is 20 for Barnase, 41 for E2/Im2 and 29 for Maxi. For Barnase, three copies of the complex are found in the crystal structure, we have only retained in the analysis those contacts that occur in all three complexes. For Maxi, chains A and B were chosen as ligand and receptor entities, respectively.

The table also reports on the computed diffusion constants and water residence times. The diffusion constant estimates are in line for Barnase and E2/Im2, and amount to 4 · 10^5^ cm^2^/s. Although these values are an overestimation with respect to experimental measurements^37^, they correspond well to earlier reported values for SPC water^38^,^39^. A lower diffusion of 2.3 · 10^5^ cm^2^/s is found for Maxi, which can be accounted for by the lower simulation temperature of 273 K. The residence times of associated water molecules lie in the order of 7 to 8 ps and agree with previous calculations^36^. In our comparison with the results of^36^, which were obtained with the TIP3 water model, we obtain the same bimodal distribution of intermolecular water angles, and we can also reproduce the particular water network structures inside the protein, so that we are confident that the differences in the water models do not play a role for our results. The lower value found for Maxi can be attributed to an increased fluidity of the environment, which is due to the presence of alanine residues in the protein, which are also accessible from the outside.

**Figure 3:**
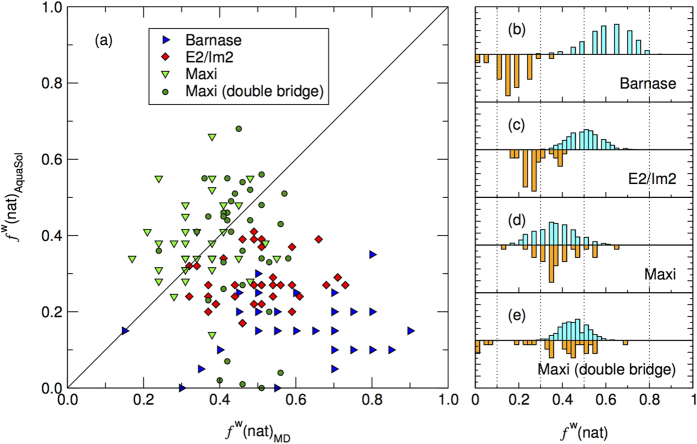
Comparison of MD simulation with AquaSol. (**a**) *f*^w^(nat) values, AquaSol *vs.* MD simulation, for the three complexes. (**a**) Barnase, blue triangles; E2/Im2, red squares; and Maxi, green triangles (single bridge) and green circles (double bridge). For the interpretation, see main text. (**b–e**) Distribution of *f*^w^(nat) values from MD (light blue) and AquaSol (orange). Dotted vertical lines delineate category of prediction quality^19^, going from *bad* (*f*^w^(nat) < 0.1) to *outstanding* (*f*^w^(nat) ≥ 0.8).

Looking more into detail at the interfacial water molecules, we use the recall of water-mediated inter-chain contacts, *f*^w^(nat), to estimate the quality of prediction. The measure is readily calculated from a single MD frame, where the water molecules can either be directly used, or *a posteriori* placed by AquaSol, giving rise to the quantities *f*^w^(nat)_MD_ and *f*^w^(nat)_AquaSol_, resp. *f*^w^(nat)_AquaSol_ values have been calculated for a representative selection of configurations, chosen at random. Those value pairs are shown exhaustively in Fig. 3a. For reasons of clarity, we discuss first the two protein complexes, and then Maxi.

### Barnase/barstar and E2/IM2

The MD results for the prediction of interfacial waters in both complexes are very good according to the evaluation scheme of ref. 19. The top panel of Fig. 3b (light blue bars) shows that the *f*^w^(nat) values for Barnase fall in the range 0.5 ≤ *f*^w^(nat) <0.8 and can therefore be termed “excellent”. For E2/Im2 (Fig. 3c), the values are slightly lower, with a distribution centering on *f*^w^(nat) = 0.5, balancing between the “good” and “excellent” categories. Such values lie a tenth of a point lower than what was found previously^19^, but it should be noted that those simulations held the relative position of the monomers fixed, whereas in our work everything was free to move. One can also assume that our simulation temperature of 300 K will have an adverse effect on *f*^w^(nat) values, as the crystal structure was solved at cryogenic temperatures (100 K).

AquaSol predictions are significantly worse for these two complexes (Fig. 3b and c, orange bars): for the barnase complex they can at best be considered as “fair”, while for E2/IM2 the best values are only found at the lower tail of the MD distribution values (“good”). The number of interfacial waters in both complexes differs slightly, between 15 for Barnase and 22 for E2/Im2. However, the number of contacts that these waters mediate is significantly different and goes from 20 for Barnase to 41 for E2/Im2. An investigation of the composition of the protein-protein interface in both complexes reveals a more polar interface for E2/Im2, with a charged/polar/non-polar ratio of 47%/24%/29%, as opposed to 34%/29%/37% for Barnase. In the more polar interface of the E2/Im2 complex, two-thirds of all water-mediated contacts involved charged or polar residues on both sides, whereas this is only half for Barnase (data not shown).

So it would seem that continuum methods show better performance as the interface becomes more polar. This is not surprising, since placement of water molecules in the AquaSol methodology occurs on the basis of electrostatic potential and the larger this potential is, the sooner a water molecule will be placed at that location. More generally stated, since in continuum methodology a more polar environment can be translated into a higher dielectric constant, the more this environment resembles bulk water, the better the performance will be.

**Figure 4:**
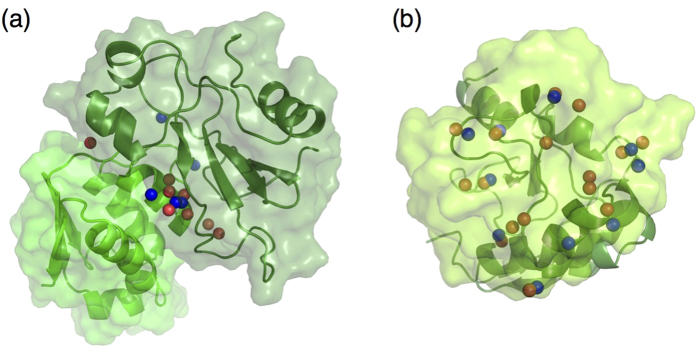
Comparison of interface-water positions generated by AquaSol (blue spheres) to those occurring in MD (red spheres). (**a**) Barnase (dark green) and barstar (light green), both plotted in surface and cartoon representation. (**b**) Top view of DNase E2 (above) in cartoon and Im2 (below) in surface representation. The *f*^w^(nat) values corresponding to these frames are 0.7 (MD) and 0.25 (AquaSol) for Barnase, and 0.73 (MD) and 0.27 (AquaSol) for E2/Im2.

The nonetheless disappointing AquaSol results are illustrated in Fig. 4a. Both protein partners are plotted in surface and cartoon representation, and there is only very little overlap between the MD (red) and the AquaSol waters (blue). Figure 4b on the other hand, nicely shows the functioning of AquaSol. The figure shows a top view of E2/Im2, showing the circumferential positioning of clusters of water molecules. For each cluster of two or three MD waters, AquaSol places one or two water molecules. Interestingly, such conservative placement was also observed in ref. 19, where an analysis of different water placement methodologies was performed and typically only one water molecule per cluster of waters was recovered.

Figure 4b also shows three water molecules at the center of the interface, which are not recovered by AquaSol. These waters represent buried water molecules. Table 2 gives a detailed look at the AquaSol predictions for the residues at the interface of the E2/Im2 complex. The Table, which follows Table S1 of ref. 19, shows that most of the water molecules that are in contact with bulk water (*i.e.* they are not buried) can at one point be recovered by AquaSol. However, the table also shows that it is much more difficult to recover contacts involving buried waters. Three of the nine buried waters can be recovered by AquaSol. These waters are essentially buried waters 7, 8, and 9 of ref. 19, which are the most solvent-exposed ones (the numbering is on increasing exposure). Buried water 2, mediating a contact between Asp-62A and Ser-74B, is only recovered because it forms a cluster with water number 9, and it is not the actual water that is recovered, but its contacts that are captured by the other water molecule. The most buried water molecules (numbers 1–6) are categorically not recovered by AquaSol. This includes buried water 5, which although buried, mediates a highly polar contact, between Asp-33A O*δ*_2_ and Arg-98B N*η*_2_.

### Maxi

Maxi has been the topic of an MD study which investigated the properties of core water molecules^36^. In our work, we revisit these findings using a different water model, SPC as opposed to TIP3P. Calculating the distribution of angles of water molecules in the protein core, we find a bimodal distribution with peaks at 10 and 46 degrees, in agreement with the values obtained previously^36^. Our simulations also reproduce the pentagonal structures of the water network, albeit not as complete as observed in the crystal structure. Revisiting Table 1, the diffusion constants of bulk water are also in accord, with values of 2.3 · 10^−5^ cm^2^/s and 2.2 · 10^−5^ cm^2^/s for ‘random’ and ‘crystal’, resp., *vs.* 3.1 · 10^−5^ cm^2^/s found by Sharp^36^. The difference can be accounted for by the chosen water model. TIP3P, the water model used by Sharp, is known to lead to faster diffusion than SPC^39^. Inside the protein core, a diffusion constant of 0.3 · 10^−5^ cm^2^/s is found (for ‘random’, and 0.4 · 10^−5^ cm^2^/s for ‘crystal’) *vs.* 0.7 · 10^−5^ cm^2^/s by Sharp, making the effect of the confinement of the water molecules in the protein core slightly more pronounced in our description. This is also reflected in the residence times of water molecules outside the core region (protein-associated waters), where we obtain a value of 7 ps compared to the value of 8 ps found by Sharp. Residence times of water molecules inside the core were found to be 14 ps on average. In the crystal structure, the number of water molecules as defined to be in the core region is 321. We find an only slightly lower amount of 296 ± 16 water molecules in this region over the course of the simulation.

Of the 321 core water molecules only 22 can be considered interfacial water molecules, and these make a total of 29 water-mediated contacts. In the crystal structure, these molecules are primarily found at the center and edges of the protein structure, as shown in Fig. 1. The recall of these water molecules can on average be called “good”, with the majority of recall values lying in the range 0.3 ≤ *f*^w^(nat) < 0.5, both for MD as well as AquaSol-predicted waters. Nevertheless, this means that 50–70% of contacts are false positives. Looking into detail at the distribution of these waters, we observed that during the course of the simulation, contact-mediating waters are distributed all along the protein, and their number increases substantially, to as many as 193 at the end of the simulation. This cannot be due to the water model, since a quick test with TIP water shows 196 of such waters at the end of a 10 ns simulation. One possible explanation could be that current water models are too tightly packed in the vicinity of proteins, or hydrophobic residues in particular, and, therefore, also at the core of Maxi, which contains a large amount of alanines.

Core waters of Maxi do not show the same behaviour as interfacial water molecules of “regular” protein complexes, but fall into an intermediate regime between interfacial and protein-associated waters, evidenced also by the extended residence time of 14 ps, which is twice as long as the residence time of protein-associated water. In order to capture the more diffuse behaviour of core water molecules, we have devised an extension of the water-mediated inter-chain contacts, called double-bridge contacts, where inter-chain contacts can be mediated by *two* water molecules. This is illustrated in Fig. 2. In the crystal structure, 95 such water molecules can be found, which are involved in 78 contacts. The MD simulation shows an average of 117 ± 17 water molecules involved in double bridge contacts, which is acceptable albeit slightly higher than the crystal structure and reflects the more compact behaviour of water, as mentioned just before.

The distribution of double-bridge *f*^w^(nat) values for Maxi, shown in Fig. 3e, shows them to lie in the range 0.3 ≤ *f*^w^(nat) < 0.6, which is an improvement over the single-contact *f*^w^(nat) values and only marginally weaker than those of E2/Im2. With some exceptions, most of the AquaSol values can be found in the same region, indicating that AquaSol is capable of recovering a great deal of the core water positions of Maxi.

### Summarizing

Our findings can be summarized in Table 3, which shows representative frames belonging to the best results obtained for *f*^w^(nat) for the three systems, for both MD simulation and AquaSol.

We conclude from the results that molecular dynamics simulation with explicit solvent is quite capable of treating the dynamics of interfacial water molecules, even though crystal water molecules in the interface had been removed prior to solvation, with later stages of the simulation revisiting the native-like initial organization of the crystal structure, as evidenced by *f*^w^(nat) values around 80% for Barnase and E2/Im2. The AquaSol *f*^w^(nat) values however, are systematically lower than the MD values, and rarely exceed 40%. Inclusion of the Yukawa potential has little to no effect on these values. For both Barnase and E2/Im2, the number of interfacial water molecules obtained by both methods is an overestimation with respect to the crystal structure, but comparable. However, the number of those molecules involved in native contacts is severely inferior for AquaSol in the case of E2/Im2, and even more so for Barnase. The slightly better performance of AquaSol on E2/Im2 is due to the more polar nature of the protein interface.

For Maxi, the story is slightly more complicated. Core waters in Maxi only contribute to a limited number of water-mediated contacts, and this number is severely overestimated in the simulations. Here lies also the reason for the high recall values, which are simply due to the high number of interfacial waters recovering contacts by chance. But the measure is not meaningless. The 22 interfacial water molecules as defined by our measure mediate true inter-chain contacts, and those contacts are probably important for the structural integrity of the protein. It is reassuring to observe that those waters show “good” recall values, both for MD and AquaSol. Our extended measure of the double-bridge contacts covers better the water molecules in the core of the protein, capturing about a third of the core waters. Also here, *f*^w^(nat) values occupy a satisfying range around 50% recall. Starting the MD simulation with the crystal water positions has little influence on the results, which had been concluded previously for barnase/barstar as well^19^.

The simulation of Maxi shows an increase in number of interfacial water molecules, which can only be due to both protein chains getting closer together. We hypothesize that this results from an underestimation of the repulsion between water and hydrophobic residues of the protein, notably alanines. Incidentally, this is likely also the reason for the “excellent” *f*^w^(nat)_MD_ recall values for Barnase. With the number of core waters being in strong agreement with the crystal structure and the protein chains finding themselves closer together, we conclude that the core waters in our simulation show too large a diffusion and are not as *ice*-like as they should be. It has been argued before that simple and local corrections to empirical force fields may significantly improve the solvation of proteins^40^, but the Maxi systems shows that particular care should be taken to ensure a proper treatment of the hydrophobic protein-water interactions.

### Conclusions

In this work we have investigated the positions of interfacial water molecules from molecular dynamics simulations and an explicit-solvent continuum model. Based on three challenging examples which reflect different types of interfacial waters, we have presented a methodology to classify these waters and quantify the prediction of water positions of the computational approaches. As a general trend, exemplified by the comparison of the results in Fig. 3, we observe that the MD-based recall values are better than those obtained from AquaSol, with the discrepancy largest for the barnase/barstar complex. Given the simulation temperature, the recall values for MD of 40–70%, labelled “good” to “excellent”, are satisfactory. For these systems, AquaSol is unable to recover buried water molecules, even when these are involved in highly polar inter-chain contacts. As one would expect from a continuum theory, the agreement with MD is best for Maxi. Here, the overall low level of prediction must be attributed to the more dynamic, ‘bulk’-like behaviour of the waters. Future work in improving in particular the continuum approach, which has the advantage of computation speed, must lie both in the development of more sophisticated water models, but also in going beyond the mean-field approximation.

## Methods

### MD simulations

The three-dimensional coordinates of the systems were retrieved from the Protein Data Bank, entries 1BRS (Barnase/barstar, chains A:D)^15^, 3U43 (E2/Im2)^19^ and 4KE2 (Maxi)^35^. Missing atoms or residues were added with the Jackal package^41^ or interactively with VMD in the case of Barnase by copying from another chain in the unit cell. The systems were prepared in an octahedron periodic box, using a minimum distance of 1 nm between the protein and the boundary. Solvation was achieved using the standard Gromacs solvate tool^42^, see also http://www.gromacs.org/. All ions and crystal waters were removed before solvation, including the interfacial water molecules. However, we also prepared simulation runs of Maxi where crystal waters were kept. Both systems are referred to as ‘random’ and ‘crystal’, respectively. Systems were made electrostatically neutral with randomly placed Na^+^ and Cl^−^ counterions. Due to its peculiar nature, the neutral system Maxi was charged at a concentration of 7 mmol/L. However, we found none of the counterions to interact significantly with the protein during the course of our simulations. MD simulations were performed with the Gromacs software^42^, v5.0.4. using the charmm27 force field^43^ and the SPC water model^44^.

Prior to data production, the systems were minimized using the steepest descent method until the maximum force on any atom was lower than 10^3^ kJ mol^−1^ nm^−1^. The systems were then equilibrated by a 100 ps *NVT* run to thermalize the system, followed by a 200 ps *NpT* equilibration run to stabilize the volume. During the equilibration, all bonds were constrained using the LINCS algorithm^45^. A time step of 2 fs was employed, Van der Waals interactions were cut off at 1 nm, electrostatic interactions were calculated with PME^46^. Equations of motion for the water molecules were solved with the SETTLE algorithm^47^. The system was coupled to a Berendsen temperature bath^48^ of 300 K (Barnase, E2/Im2) or 273K (Maxi), with protein and solvent coupled independently. Pressure was maintained at 1 bar by the Parrinello-Rahman barostat^49^. Data production runs consisted of 10 ns simulations, with bond constraints only on bonds involving hydrogen atoms. The configuration of the system was saved every 10 ps.

### Diffusion and residence times

In order to calculate diffusion rates of water molecules, additional short simulations were performed for 300 ps, saving the configuration every 200 fs. The diffusion constant was computed by calculating the mean square displacement of a selected number of water molecules and then using the Einstein relation, using the Gromacs suite of analysis tools. In order to calculate the residence times of protein-associated water molecules, we monitor the total time a first-shell water molecule stays in contact with a protein atom. Any water (oxygen) within a distance of 3.5 Å from a protein heavy atom was considered to be in contact with the protein. This is a slightly simplified approach as employed in ref. 36, since we do not distinguish between different atom types. The residence time is then calculated by averaging over all residence times of associated water molecules.

### AquaSol

We obtained the AquaSol software^27^ from the authors and installed it locally. We use both the basic DPBL-model as well as the Yukawa functional which takes repulsion between the water molecules into account^50^. Conversion from pdb to pqr format was done using the online pdb2pqr Server^51^. The simulation temperature was the same as for the MD simulation (300 K and 273 K) and the implicit ion concentration was set to match the volume and number of ions in the MD simulation box. The number of points in the *x*, *y* and *z* dimensions (2^*n*^ + 1) was 129 × 129 × 129 for Barnase and E2/Im2, and 65 × 65 × 129 for Maxi. This ensures a resolution of around 1 Å in each dimension. Individual coordinate configurations, chosen at random, were extracted from the MD runs and used as input structure in the AquaSol software^27^. The configurations with the best *f*^w^(nat)_MD_ values are listed in Table 3. A dielectric constant of 3 was employed for the protein interior. We have tested the effect of the chosen value on several residues in order to see how the choice affects the obtained *f*^w^(nat) values. For all residues tested the general trend is observed that for a smaller value (*ε*_*p*_ = 2) the *f*^w^(nat) -values increase while they decrease for a larger value (*ε*_*p*_ = 4), as a consequence of the increased dielectric contrast between the protein and the surrounding solvent for the lower dielectric constant of the protein. We opted for the value of 3 as a compromise on the usual scale of *ε*_*p*_ ≈ 2−4 and performed all analyses for this value. The lattice grid size for the solvent was 2.8 Å with a concentration of 55 mol/l and dipole moment of 3.0 debye. Placement of water molecules was done using the method described in the paper of Azuara *et al*.^26^ which consists of sorting the density values in descending order. The water molecules are then placed by walking down the list until the density threshold has been reached, which in our case was the reference density of bulk water. At every water molecule placement we eliminate points within 3.0 Å of this position from the list.

## Additional Information

**How to cite this article**: Copie, G. *et al*. On the ability of molecular dynamics simulation and continuum electrostatics to treat interfacial water molecules in protein-protein complexes. *Sci. Rep.* **6**, 38259; doi: 10.1038/srep38259 (2016).

**Publisher’s note:** Springer Nature remains neutral with regard to jurisdictional claims in published maps and institutional affiliations.

## Supplementary Material

Supplementary Material

## Figures and Tables

**Table 1:** Initial and computed characteristics of the MD simulations of the three systems.

System	Barnase	E2/Im2	Maxi
Protein atoms	3159	3612	4760
Water molecules	15659	11298	11662
Ions	4 Na^+^	4 Cl^−^	2 Na^+^, 2 Cl^−^
Box size (nm^3^)*	7.9 × 7.9 × 7.9	6.6 × 10.1 × 5.7	4.7 × 4.6 × 17.8
Temperature (K)	300	300	273
Interfacial waters^a^	15	22	22
Water-mediated contacts^a^	20	41	29
Diffusion coefficient in bulk^b^	3.8	4.1	2.3
Residence time of associated water	8 ps	8 ps	7 ps

^a^Values change over the course of the simulation, initial values reported here.

^b^Diffusion constant × 10^5^ cm^2^/s.

**Table 2:** Table listing the water-mediated ligand-receptor contacts of the E2/Im2 complex, for the frame corresponding to Fig. 4a.

	Ile22A	Cys23A	Arg24A	Glu26A	Gly27A	Glu30A	Glu31A	Asp33A	Asn34A	Arg38A	Glu41A	Ser50A	Asp51A	Ile53A	Tyr54A	Tyr55A	Pro56A	Asp58A	Asp62A
Gln 70B																**W**			
Lys 72B															**B1/6**	**W**	*W*	*W*	
Gly 73B														**B2**					*B2*
Ser 74B														**B2**	**B1**				*B2/9*
Asn 75B															**B1/6**				
Thr 77B		*W*																	
Asn 78B	**B4**							**B5**							**B4**				
Lys 81B			*W*	*W*															
Gly 82B						*W*													
Lys 83B					**W**	*W*													
Ala 87B												**B3**	**B3**						
Arg 88B													*W*						
Lys 89B													*W*						
Lys 90B													*W*						
Gln 92B											**B7**	*B7*	**B3**						
Gly 95B							*W*		*W*										
Glu 97B									*W*	*B8*									
Arg 98B						*W*		**B5**	*W*										

Residues involved in such contacts show a **W** at their intersection, and a **B** if the water molecule responsible for the contact is buried. The table and the numbering of the buried waters follow Table S1 and Fig. 1 of ref. 19, resp. Waters that are recovered by AquaSol are listed in Italics.

**Table 3:** Values of the *f* ^w^(nat) coefficient calculated from MD and AquaSol-predicted water positions of selected simulation frames.

		*f*^w^(nat			Number of water molecules			
**Barnase**		**MD**	**PBL**	**Yukawa**	**AquaSol**	**MD**		
	*t* = 3.8 ns	0.85	0.15	0.15	16 (3)	17 (8)		
	*t* = 8.1 ns	0.80	0.30	0.30	16 (6)	22 (10)		
**E2/Im2**		**MD**	**PBL**	**Yukawa**	**AquaSol**	**MD**		
	*t* = 1.8 ns	0.73	0.27	0.27	31 (10)	27 (17)		
	*t* = 2.5 ns	0.71	0.29	0.29	27 (11)	27 (18)		
**Maxi**		**MD**	**PBL**	**Yukawa**	**AquaSol**	**MD**	**AquaSol**	**MD**
	*t* = 0.5 ns	0.41	0.34	0.34	42 (9)	28 (10)	323	303
	*t* = 5.3 ns	0.62	0.38	0.38	56 (8)	45 (15)	359	304
	*t* = 10.0 ns	0.66	0.38	0.38	59 (9)	36 (13)	194	224
					Interface		Core	

The selected frames are hand-picked and correspond to some of the best values obtained for MD. The number of interface (and core for Maxi) water molecules is also listed, with the number of these molecules involved in native contacts in parentheses.
