# Supplementary material for: On the ability of molecular dynamics simulation and continuum electrostatics to treat interfacial water molecules in protein-protein complexes
Source: Sci Rep. 2016 Dec 1;6:38259. doi: 10.1038/srep38259 (PMC5131287; doi:10.1038/srep38259)
Supplement: Supplementary Material [file srep38259-s1.pdf]

Supplementary Material for

'On the ability of molecular dynamics simulation and continuum electrostatics to treat interfacial water molecules in protein-protein complexes'

Guillaume Copie[1,2], Fabrizio Cleri[1], Ralf Blossey[2,*] and Marc F. Lensink[2,*]
[1]University Lille, CNRS, UMR8520 IEMN, Lille, F-59000, France
[2]University Lille, CNRS, UMR8576 UGSF, Lille, F-59000, France

Convergence of MD simulations for f^w(nat):

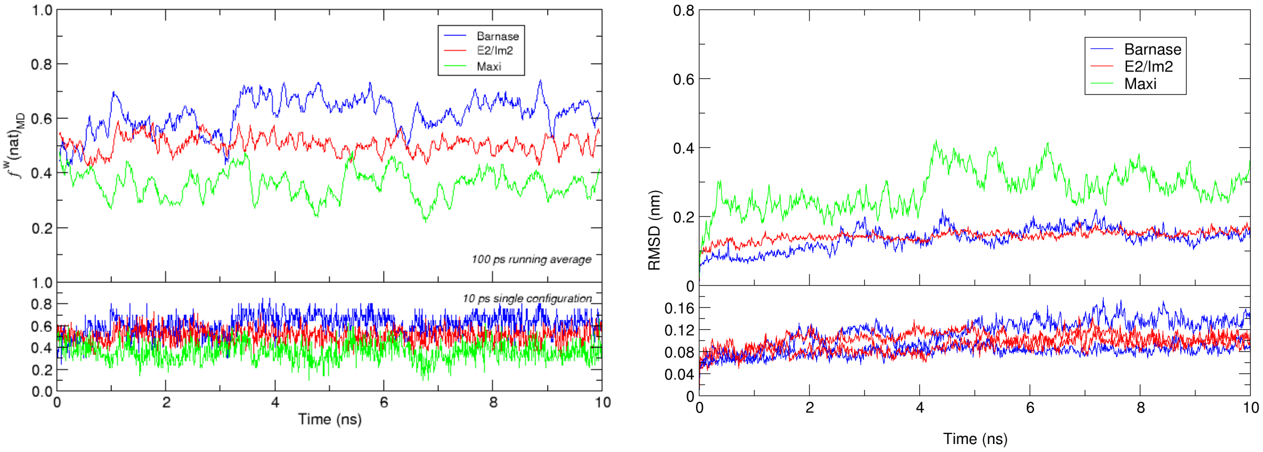

Supplementary Figure 1 – (a, left) Evolution of the calculated f^w(nat) values over the course of the molecular dynamics simulations. The bottom panel shows the individually calculated values, while the top panel shows the running average of these over a 100-ps window. (b, right) C-alpha root mean square deviation (RMSD) of the protein complexes (top panel) and individual subunits (bottom panel) over the course of the simulations.

The goal of this work is to look at how well MD and continuum electrostatics behave in the perfect situation where the proteins are at their optimal position. For lack of a better reference, this can only be the crystal structure. Longer simulations tend to move away from that situation. This is the case for a single protein and even more so for two non-covalently bound molecules in a protein complex. Supplementary figure S1b (right-hand figure) shows the evolution of the root mean square deviation (RMSD) over the course of the MD simulations. The figure nonetheless illustrates the stability of the complexes (top panel) and individual subunits (bottom panel, the Maxi protein is an obligate dimer and hence not shown). The stability is also evident from Supplementary Figure S1a (left-hand figure above), which shows the calculated f^w(nat) values of the simulations. MD snapshots are taken and analyzed every 10 ps, which properly samples the dynamics of the water molecules. For a subset of configurations, chosen at random, AquaSol values have been calculated. For the two systems where the notion of interfacial water molecules is most important, Barnase and E2/Im2, AquaSol values are categorically below MD values. This is discussed in the main text.

2. Use of SPC vs TIP3P

We have reproduced the simulation results by Sharp (ref. [36]) on Maxi in order to assure ourselves that the results did not depend on the chosen water model. Supplementary Figures 2 and 3 illustrates that our simulations reproduce well both the bimodal distribution of water-water H-bond angles

(Figure 8 in Sharp [36]) as well as the details of the water network, in particular the pentagonal rings, evidenced in the original paper on the structure of Maxi (ref. [35] of the paper).

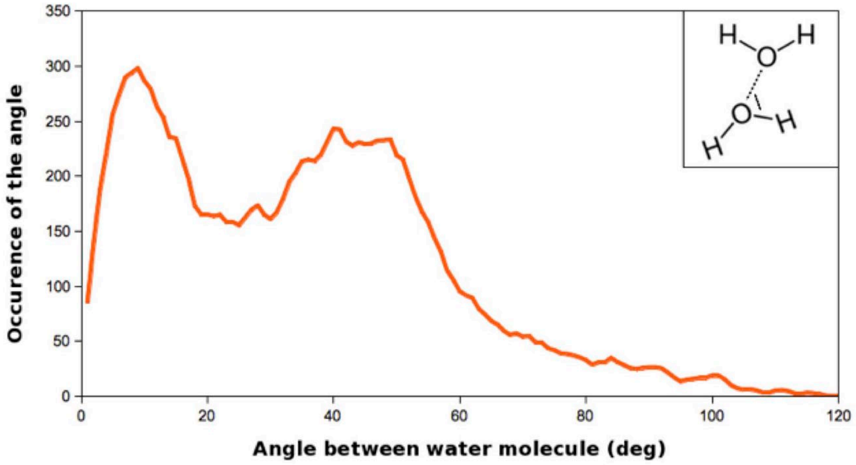

S2: water-water H-bond angle distribution

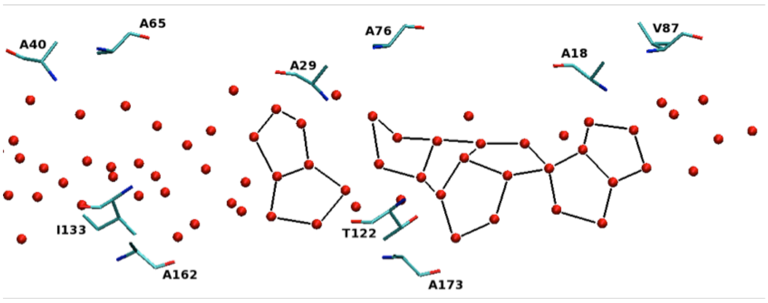

S3 ring structures in Maxi interior
